# Are hypopressive and other exercise programs effective for the treatment of pelvic organ prolapse?

**DOI:** 10.1007/s00192-022-05407-y

**Published:** 2022-11-23

**Authors:** Kari Bø, Sònia Anglès-Acedo, Achla Batra, Ingeborg H. Brækken, Yi Ling Chan, Cristine Homsi Jorge, Jennifer Kruger, Manisha Yadav, Chantale Dumoulin

**Affiliations:** 1 Department of Sports Medicine, The Norwegian School of Sport Sciences, PO Box 4014, Ullevål stadion, 0806 Oslo, Norway; 2 Department of Obstetrics and Gynecology, Akershus University Hospital, Lørenskog, Norway; 3 Urogynaecology Unit Hospital Clínic de Barcelona, Barcelona, Spain; 4 Department of Obstetrics & Gynaecology, VMMC & Safdarjung Hospital, New Delhi, India; 5 Kolbotn Physical Institute, Nordre Follo Municipality, Norway; 6 The Pelvic Floor Centre, Division of Surgery, Akershus University Hospital, Lørenskog, Norway; 7 Department of Obstetrics and Gynaecology, Calderdale and Huddersfield NHS Foundation Trust, Huddersfield, UK; 8 Department of Health Science Ribeirão Preto Medical School, University of São Paulo, São Paulo, Brazil; 9 Auckland Bioengineering Institute, University of Auckland, Auckland, New Zealand; 10 Paropakar Maternity and Women’s Hospital, Kathmandu, Thapathali Nepal; 11 School of Rehabilitation, Faculty of Medicine, Université de Montréal, Montréal, Canada

**Keywords:** Exercise, Physical therapy, Hypopressive, Kegel, Pelvic floor muscle training, Pilates

## Abstract

**Introduction and hypothesis:**

Pelvic floor muscle training (PFMT) is effective for the treatment of pelvic organ prolapse (POP), but other exercise programs have also been promoted and used. The aim of this review was to evaluate the effect of hypopressive and other exercise programs besides PFMT for POP.

**Methods:**

A literature search was conducted on Ovid Medline, EMBASE, CINAHL, Cochrane, PEDro, and Scopus databases from January 1996 to 30 December 2021. Only randomized controlled trials (RCTs) were included. The keywords were combinations of “pelvic organ prolapse” or “urogenital prolapse,” and “exercise therapy,” “hypopressive exercise,” “Kegel,” “pelvic floor muscle training,” “pelvic floor muscle exercises,” “Pilates,” “treatment,” “yoga,” “Tai Chi.” Methodological quality was assessed using the PEDro rating scale (0–10).

**Results:**

Seven RCTs containing hypopressive exercise, yoga or breathing and hip muscle exercises in an inverted position were retrieved and analyzed. PEDro score ranged from 4 to 7. There was no additional effect of adding hypopressive exercise to PFMT, and PFMT was more effective than hypopressive exercise alone. The studies that included the term “yoga” included regular PFMT and thus can be classified as PFMT. Hip exercises in an inverted position added to PFMT vs PFMT alone showed better improvement in some secondary outcomes but not in the primary outcome, POP stage.

**Conclusions:**

There are few RCTs assessing the effects of other exercise programs besides PFMT in the treatment of POP. To date, there is no evidence that other exercise programs are more effective than PFMT for POP.

## Introduction

To date there has been a grade A recommendation based on level 1 evidence on pelvic floor muscle training (PFMT) being an effective treatment in reducing symptoms and stages of pelvic organ prolapse (POP) [1, 2]. Understanding of how and why PFMT is effective in the treatment of both stress urinary incontinence (SUI) and POP is based on the anatomical, biomechanical, and exercise science rationale for actions during a single PFM contraction and the effects of doing regular PFMT over time [3, 4]. A single voluntary contraction of the PFM has been shown to reduce the levator hiatus (LH) area by 25% from a resting area of 20 cm^2^ (95% CI 17 to 23) to 15 cm^2^ (95% CI 13 to 17), and the muscle length shortens 21% from 12.5 cm (95% CI 11.1 to 13.8) to 9.7 cm (95% CI 8.7 to 10.7), thereby closing and tightening the pelvic openings [5]. In addition, the PFM is lifted higher up in the pelvis, stabilizing the pelvic floor [5, 6].

One approach, termed “the knack”, is to practice pre-/co-contraction of the PFM during situations where such contractions are needed to prevent excessive opening of the LH area and downward movement [6–8]. The effect of regular PFMT over time has been shown to induce morphological changes to the pelvic floor directly addressing the underlying mechanisms of POP. In an assessor-blinded RCT involving 109 women with Pelvic Organ Prolapse Quantified System (POP-Q) stage I, II, and III, Brækken et al. [9], using 3D and 4D perineal ultrasonography, found that 6 months of PFMT caused morphological improvements of the pelvic floor. These changes were reflected in elevation of the bladder neck and rectal ampulla by 4.3 mm and 6.7 mm respectively, narrowing of the LH area by 1.8 cm^2^, increased PFM thickness by 16% and reduced muscle length by 4%. In this trial, there was also less opening of the LH area and reduced increase in muscle length during straining in the PFMT group, which may indicate improved automatic function and increased PFM stability [9].

In parallel to the anatomical, biomechanical, and exercise science rationale and evidence from RCTs and systematic review of PFMT for POP [1, 2], there are suggestions in the literature that other exercise programs or techniques may have an equal or better effect than PFMT [10, 11]. In addition, there is significant and recurrent exposure of other exercise programs or techniques, especially hypopressive exercise, on social media channels (such as YouTube, Instagram, and TikTok) advertising and selling exercise programs to treat POP [12]. Some even claim that PFMT is ineffective in treating POP, again without references. The aims of the present review were to evaluate the effect of performing other exercise programs or techniques besides or as an alternative to PFMT for symptoms and stage of POP, and whether these exercise programs are more effective than PFMT alone.

## Materials and methods

In this narrative review, Ovid Medline, EMBASE, CINAHL, Cochrane, PEDro, and Scopus databases were searched between January 1996 and December 2021 to identify studies on the most well-known popular exercise programs. The keywords were combinations of “pelvic organ prolapse” or “urogenital prolapse,” and “exercise therapy,” “hypopressive exercise,” “Kegel,” “pelvic floor muscle training,” “pelvic floor exercise,” “Pilates,” “posture,” “treatment,” “yoga,” and “Tai Chi.” An updated search was conducted on PubMed in January 2022. A limitation was set on fully published randomized controlled trials (RCTs) or full publication RCTs in languages other than English with at least an English language abstract. Results from trials reported in abstracts only were excluded. We did a manual search for publications from the International Consultation on Incontinence (ICI) [1], NICE (2019) [13] and the book chapter on PFMT for POP by Bø and Frawley [14].

The PEDro rating scale [15, 16] was applied to assess risk of bias (internal validity) for the RCTs. Table 1 shows the ten criteria used in this rating scale. Criteria A (eligibility) concerns external validity (generalizability). A total PEDro score equal to or less than three points is considered poor, a score from four to five is considered fair, a score from six to eight is considered good, and a score of nine to ten is considered excellent [17]. The PEDro scale has been found to be a reliable and valid tool for evaluating methodological quality in clinical trials [15, 18, 19].

**Table 1:** PEDro quality scores of randomized controlled trials

Score	Criteria	
E	Eligibility criteria specified	
1	Subjects randomly allocated to groups	
2	Allocation concealed	
3	Groups similar at baseline	
4	Subjects blinded	
5	Therapist administering treatment blinded	
6	Assessors blinded	
7	Measures of key outcomes obtained from > 85% of subjects	
8	Data analyzed by intention to treat	
9	Statistical comparison between groups conducted	
10	Point measures and measures of variability provided	

Methods, definitions, and units conform to the standards jointly recommended by the International Urogynecological Association and the International Continence Society except where specifically noted [20–22].

## Results

Table 2 shows the RCTs reporting the use of exercise regimens other than PFMT for treating POP. We found no RCTs addressing tai chi or Pilates. One study did specific PFMT as part of a Pilates class in addition to PFMT home training [23]. This study was presented and classified as PFMT by the authors and the results have been reported elsewhere [2]. Four studies (two reporting data on different outcome measures from the same study) compared PFMT with hypopressive exercise [10, 24–26]. Two RCTs reported comparing yoga with conventional exercise or usual care [11, 27] and one conducted a program including hip and breathing exercises in a specific “inverted” position in addition to PFMT compared with PFMT alone [28]. Most studies included stage II POP, with only Gorji et al. [28] also including stage III POP. Outcomes used were signs using the POP-Q system and validated instruments for symptoms and/or quality of life. PFM variables were assessed by vaginal palpation [10, 11, 24, 25, 27], surface electromyography (sEMG) [,^10^, ^24^, ^25^], manometry [26, 28], dynamometry [26], and ultrasound [25].

**Table 2:** Evidence for exercise programs other than pelvic floor muscle training for treating pelvic organ prolapse

	Design	POP	Exercise intervention	Drop-out/adherence	Outcome	Results	PEDro score (0–10)
Bernardes et al.; Resende et al. [24, 25], Brazil	Assessor blinded RCT/63 women, mean age 55.4 (SD 9.8)	POP-Q stage II	12 weeks intervention with three individual sessions of group 1 and 2. 1. PFMT(*n*=21). 2. Hypopressive (*n*=21) 10 repetitions of hypopressive exercise in association with voluntary PFM contractions. 3. Control (*n*=16): single visit with PT, learned to contract the PFM, asked to do the “Knack”. Home training for the study period with three sets of 8–12 maximal PFM contractions per day, similar time in groups 1 and 2 with two monthly visits with PT. Standard lifestyle advice for all three groups	Drop-out: *n*=5; 7.9% in the control group. Adherence (exercise diary): PFMT: 71.4%. Hypopressive + PFM contraction: 76.2% performed all exercises	*CSA measured with 2D transperineal ultrasonography [24]. *Modified Oxford grading [25]. *sEMG [24]	Change in CSA between both treatment groups compared with controls. No difference between group 1 and 2. Improvement in PFMT: 50%. Improvement in hypopressive + PFMT: 20%. Both exercise groups superior were to the control group in strength, endurance and activation. PFMT alone superior to hypopressive with PFM contractions in endurance. No effect of adding hypopressive to PFMT. No long-term data reported	Bernardes et al.: 4; Resende et al.: 6
Resende et al. [10], Brazil	Assessor-blind RCT/70 women, mean age 55.7 years (SD 5.2)	Symptomatic POP-Q stage II. 68% had anterior vaginal wall POP, 9.8% posterior and 21.3% combination	12-week intervention. All participants had three sessions with PT to learn how to do PFMT/hypopressive. Then 3 months of either 1. PFMT (*n*= 35) or 2. Hypopressive exercise (*n*=35). Both groups exercised at home with phone calls every week and individual appointments bimonthly with PT. Both groups had global stretching and lifestyle advice on weight loss, fluid intake, constipation, avoidance of heavy lifting. No voluntary contraction of the PFM during hypopressive	Drop out: 4 in hypopressive, 5 in PFMT. Adherence (exercise diary): hypopressive: 89% PFMT: 84%	Primary: *P-QoL. Secondary: *POP-Q *Modified Oxford grading *sEMG	All measures significantly better in PFMT: Symptoms: effect size: 1.01 (95% CI: 1.002–1.021). 19 (67%) of anterior prolapse lifted 1 stage, 4 (45%) of posterior prolapse lifted 1 stage after PFMT. Significant difference in favor of PFMT vs hypopressive in anatomic POP. Urinary and bowel symptoms: favor of PFMT: SUI (*p*<0.001), straining to empty bladder (*p*<0.001), vaginal bulge interfering with emptying of the bowel (*p*=0.002). No long-term data reported	6
Navarro-Brazález et al. [26], Spain	Assessor blind RCT *N*= 94, mean age 47 (SD 10)	Women with PFD: SUI, MUI, and /or POP (stage I or II); 45.7% had POP stage I or II. 1.PFMT group: 40.6% 2.Hypopressive: 35.5% 3.PFMT + hypopressive: 61.3%	8 weeks intervention. 2 visits of 45 min each week of: 1.PFMT (*n*=32) (+ e-stim if score < 3). 10 maximal contractions + rapid, holding time 10 s. Home exercise 2–3 sets of 5–10 PFMc/day. 2. Hypopressive + home hypopressive (*n*= 31). 3.PFMT + hypopressive (*n*=31). All participants had evaluation of correct PFM contraction and instruction in “the Knack”. Hypopressive exercise without voluntary PFMc	Drop out: 2 + 3 for follow-up. Adherence (asked by PT) to home exercise: 1.PFMT: 71.9%; 2.hypopressive: 61.3%; 3.PFMT + hypopressive: 67.7% Adherence fell below 60% in all groups at 6 months with no difference between groups. Knack incorporated into activities of daily living in 85% during the intervention, no difference between groups. Use of Knack at 12 months: PFMT: 68.8%; hypopressive: 83.9%; PFMT + hypopressive: 80.6%	*Overall report of PFD assessed with PFDI-20 and PFIQ-7 *Subscores of POP *PFM variables (manometry and dynamometry)	No difference between groups in any variables. Long-term at 6 and 12 months after cessation of the intervention: effects within groups reported to be maintained, no difference between groups. Adverse effects reported as low back pain in some postures by 1 woman in PFMT + hypopressive and in 5 in hypopressive group	7
Sweta et al. [27], India	Assessor blinded RCT *N*=50, age 20–60 years (mean age cannot be reported)	Symptomatic “mild” POP	12 weeks intervention. Follow-up once per month. 1.Yoga (*n*=25): conventional treatment exercise + yoga class including + 8–10 PFM contractions twice day for 5–7 min. 2. Control (*n*=25): “conventional treatment modalities”	Drop-out: none. Adherence: not reported	*PFDI-20; *PFIQ-7; *Vaginal palpation of PFM	Moderate/severe perineal pain from 100% to 72% with mild or absent of pain in “yoga” group (*p*.<0.001). NO change in the control group. Nonsignificant change in mean 5.7 (95% CI: 3.1 to 14.7) points on the PFDI-20 in “yoga group” than the control group (*p* = 0.1). No clinical meaningful differences after yoga. Number of women with a feeling of something coming out significantly improved in the yoga group, not in the controls. No report of long-term effect	4
Gorji et al. [28], Iran	Assessor blinded RCT *N*=40. Mean age: 51.8 years (SD 11.1)	Stage II (77.5%) and III (22.5%) POP (anterior and/or posterior POP)	4-week intervention with three sessions/week. Intervention (*n*=20): PFMT+ postural or positional inversion exercise (lying on inverted wooden wedge doing exercise with the hips elevated), 10 repetitions of 4 respiration and hip exercises. Control (*n*=20): PFMT (not described)	Drop-out: 0. Adherence: not reported	Primary: POP stage (POP-Q). Secondary: QoL (P-QOL), bladder symptoms (ICIQ-FLUTS). Independent variable: PFM strength by manometry	No difference in POP-Q between groups (*p*=0.414).. General health (*p* = 0.010), physical limitation (*p* = 0.038), social limitation (*p* = 0.010) in favor of intervention. ICIQ-FLUTS: urinary symptoms: better improvement in filling symptoms, nocturia, urgency, and bother scales for women in PFMT + postural. No significant difference in frequency, SUI, and hesitancy symptoms. PFM strength in favor of adding postural/positional exercise (*p*=0.04 after intervention). No report of long-term effect	7
Sweta et al. [11], India	Nonblinded prevention RCT, *n*=54, 20–60 years of age	Healthy women with no PFD not searching help	12-week intervention. Vaginal palpation to confer correct contraction, 50 women randomized to: 1. Mula Bandha yoga therapy (*n*=25) focusing on PFM with 10 contractions twice a day in group sessions; 2. control (*n*=25): usual self-care	54 (4 excluded or did not fulfill inclusion criteria). Drop-out: 0. Adherence: not reported	PFM variables by PERFECT	No significant difference between groups in any scores of PERFECT. No report of long-term effect	5

*CSA* cross-sectional area, *ICIQ-FLUTS* International Consultation on Incontinence Questionnaire Female Lower Urinary Tract Symptoms, *PERFECT* (*P* = power, *E* = endurance, *R* = repetitions, *F* = fast contractions, *ECT* = every contraction timed), *PFD* pelvic floor dysfunction, *PFDI-20* Pelvic Floor Distress Inventory Short Form, *PFIQ-7* Pelvic Floor Impact Questionnaire Short Form, *PFM* pelvic floor muscle, *PFMT* pelvic floor muscle training, *PFMc* pelvic floor muscle contraction, *PT* physical therapist, *POP-Q* pelvic organ prolapse quantification system, *P-QoL* prolapse quality of life, *RCT* randomized controlled trial, *SD* standard deviation, *sEMG* surface electromyography, *SUI* stress urinary incontinence

PEDro scores for the included studies ranged between 4 and 7. The number of participants in each of the comparison groups ranged between 16 and 35, and the numbers were not always equal in each group. Drop-out was generally low and adherence above 70% in all RCTs. Some studies only reported outcome on PFM variables or morphometry [11, 24, 25] and not on stage of POP or on POP symptoms (Table 2).

Duration of the exercise period varied between 4 and 12 weeks and only one study reported long-term effects 6 and 12 months after cessation of the exercise period [26]. All studies included teaching and assessment of ability to contract the PFM for all participants before beginning the actual training programs. Several of the studies included PFM contractions in their alternative training programs [11, 24, 25, 27, 28]. All programs included supervised training once a week or month, and the training was combined with home training in all studies.

### “Hypopressive technique”

The “hypopressive technique” was developed by Caufriez in the 1980s [29] and involves a breathing technique suggested to lead to “low pressure”. Decreased intra-abdominal pressure (IAP) is hypothesized to lead to reflex activity of the muscles in the abdominal wall and pelvic floor, thereby reducing UI and POP [29]. In addition to the suggestion that the hypopressive technique is effective in the treatment of UI and POP, it is also advocated to be effective in the treatment of diastasis recti abdominis as well as low back pain [30].

Resende et al. [24] reported that both PFMT and PFMT plus the hypopressive technique were significantly more effective than lifestyle advice in increasing PFM strength (Oxford grading) and PFM activation (sEMG); however, there were no additional effects of adding the hypopressive technique to PFMT on either strength or activation. Ultrasound assessment of the cross-sectional area (CSA) of the levator ani muscle in the same study showed increased CSA in the PFMT and the PFMT plus the hypopressive technique compared with the lifestyle group, but no additional effect of use of hypopressive technique on CSA [25].

Resende et al. [10] found that PFMT was superior to hypopressive technique in the treatment of POP, assessed both as symptoms and stage of POP. In a mixed prevention and treatment study (including women with and without POP), Navarro-Brazález et al. [26] compared 8 weeks of PFMT with hypopressive exercise or hypopressive exercise + PFMT and found no statistically significant differences between groups after the intervention in overall report of PFD (stage of POP and POP symptoms not reported separately) or PFM variables. An adverse effect of low back pain was only reported in women who did the hypopressive exercise program.

### “Yoga”

The two studies reporting on yoga conducted conventional PFMT in the yoga classes and the participants did the same PFM exercises at home [11, 27]. This program was compared with conventional exercises or usual care. One study reported the effect this approach had on POP symptoms [27] and found the significant effect in favor of the “yoga” group did not meet the previously defined clinically meaningful difference in PFDI-20. Perineal muscle laxity was significantly reduced in the yoga group only. No effect was found on the subgroup scale of PFIQ-7. The other study [11] assessed PFM variables but not POP symptoms or stage of POP. No significant differences were found between groups in any variables.

### “Breathing exercises and exercises for the hips in an inverted position”

Gorji et al. [28] conducted an RCT comparing PFMT alone with PFMT + breathing exercise and exercises including external rotation, adduction, and abduction of the hips in an inverted position. The inverted position is explained as “positioning a subject with hips elevated on a wedge higher than the chest”. The wooden wedge is described as having a height of 15.24 cm, a width of 45.72 cm and a length of 53.34 cm. The results showed no difference between the PFMT alone and PFMT + inverted exercise in the primary outcome (POP stage). There was statistically significant difference in favor of PFMT + inverted exercise in secondary outcomes such as general health, physical and social limitation, and some urinary symptoms, but not in SUI, frequency or hesitancy symptoms. PFM strength, measured with manometry, was significantly better in the PFMT + inverted exercise group.

## Discussion

We found only 7 RCTs reporting on other exercise programs or techniques besides PFMT to treat POP, including hypopressive exercise, yoga, and inverted position hip exercise. There were no RCTs of popular exercise programs such as Tai Chi and Pilates. The trials had fair (3 trials) to good (4 trials) methodological quality, they were relatively small, but there were few dropouts and high adherence. The lack of RCTs contradicts the significant activity on social media platforms promoting these and other exercise programs as effective approaches in the treatment of POP without robust evidence [12]. The proposed mechanisms of how these alternative exercise programs can be effective at treating POP can be divided into:

1. Those that are thought to reduced IAP (hypopressive and inversion exercises)
2. Programs that are thought to indirectly activate the PFM (yoga, Pilates)
3. Those that are thought to do both (hypopressive exercise)

Overall, there were no convincing results of any of the exercise programs evaluated in the published RCTs.

### Hypopressive exercise

The effect of hypopressive exercises has been debated previously owing to a lack of evidence from high-quality RCTs [31, 32]. Resende et al. [10] showed that PFMT was superior to hypopressive exercise in all outcomes. This corresponds with the results of the same groups’ former publications finding no additional effect of adding hypopressive exercise to PFMT [24, 25]. The study of Navarro-Brazáles et al. [26] found no difference in any outcome between PFMT, hypopressive, and a combination of the two exercise programs, and concluded that they were equally effective. However, the education part of the program included information on lifestyle change and instruction of a correct PFM contraction, and the participants were advised to pre-contract the PFM before and during increases in IAP (performing the “knack”). This may explain the results just as well as the hypopressive technique. In addition, only 45.7% of the participants demonstrated POP stage I or II, and the number of women with POP was different from each of the intervention groups, yet results were reported for the overall sample with no specific analysis for the subgroup of women with POP.

The effect of hypopressive exercise is hypothesized to be caused by a reduction in IAP [33]. However, as far as we can ascertain, an effect of reduction of IAP has not been demonstrated, and if it occurs during one single maneuver, there is no evidence that this possible reduction in pressure translates to activities in everyday life. It has also been suggested that the hypopressive maneuver activates the PFM contraction [26]. In two nonrandomized experimental studies, the immediate effect of either PFM contraction or hypopressive technique on the PFM was investigated. Resende et al. [24] assessed the acute effect of a PFM contraction, the hypopressive technique, and a combination of the two. Thirty-six nulliparous physical therapists were examined with vaginal sEMG during the three maneuvers. The results showed that PFM contraction was more effective than the hypopressive technique at increasing sEMG activation of the PFM and that there were no additional effects from adding the hypopressive technique. The hypopressive technique was significantly more effective than PFM contraction in activation of the transverse abdominal muscle [24]. Resende et al. [34] measured the LH area in 17 nulliparous women with 4D ultrasonography. During PFM contraction the reduction of the LH area was 1.8 cm^2^ and during the hypopressive technique and hypopressive + PFM contraction 0.5 cm^2^ and 2.0 cm^2^ respectively. They concluded that there was no statistically significant reduction in LH area in healthy nulliparous women by using the hypopressive technique or adding PFM contraction to the technique. A short-term experimental study of Navarro-Brazáles et al. concluded that the activation levels (sEMG) of the PFM and abdominal muscle during hypopressive exercise is likely insufficient to result in strength gains, but they could have an endurance effect [35]. In a more recent abstract from the ICS using the FemFit© measuring PFM contraction and IAP simultaneously, it was found that the mean peak pressure was > 80% higher during a voluntary PFM contraction than during two different hypopressive techniques [36]. Only a maximum voluntary PFM contraction produced higher vaginal peak pressures than those observed at rest. There was no reduction in IAP during the hypopressive technique, again questioning the theoretical rationale for the technique [36]. There is a need for more basic research and high-quality RCTs on the mechanisms and possible effects of hypopressive exercise.

### Yoga

There are a number of issues with the RCTs on yoga [11, 27]: the inclusion criteria for POP were unclear, the conventional treatment was not described, and the description of the yoga practice was equivalent to a PFMT program. The “yoga group” performed PFM contractions 8–10 times for 5–7 min twice per day for 12 weeks. Hence, we would classify this as a PFMT program and not as general yoga exercises, and the study cannot be used as evidence for yoga as such in the treatment of POP. Effective PFMT has been conducted within a group training concept for many years [4, 37] and can be included in any general exercise program if they are conducted separately from other exercises. There is strong evidence that group training of the PFM works for SUI [4, 37–39], and that group training is not inferior to individual training for SUI [38]. In the trials of PFMT for POP only two trials were conducted within a group training concept [23, 40]. Due et al. [40] did not find any effect on the primary outcome, stage of POP, but found a significant effect on POP symptoms in their study, whereas Hagen et al. [23] found a small but statistically significant effect of PFMT included in a Pilates class + home PFMT. To date, there have been no “head-to-head” RCT comparison studies on the effect of individual versus group training for POP. Based on the current evidence, individual PFMT is recommended. Whether PFMT within a yoga class setting is effective at preventing or treating POP remains to be investigated in a high-quality RCT.

### Breathing and hip exercises in an “inverted position”

Gorij et al. [28] did not find any effect on their primary outcome (POP stage by POP-Q system) of adding inverted position with breathing and hip exercises to PFMT. There was, however, a statistically significant effect on some, but not all, of the secondary outcomes and PFM variables. The exercise program was of short duration, with only 4 weeks, compared with the 12 weeks in the other studies included in this review. In addition, the group with exercises in the inverted position involved longer treatment duration and more supervision from the provider of the intervention, which may account for the finding that PFMT was inferior to the new concept in some of the measurements. All women in the “new” exercise program performed the exercises in an inverted position, and although it is possible that inversion of position may decrease IAP and reposition the prolapse, it is not possible to conclude which of these two interventions (inversion or hip and breathing exercise) may be associated with an effect. It is difficult to see the rationale for why exercises for hip adduction, abduction, and external rotation and breathing should have any effect on POP, except for a possible co-contraction of the PFM. Such co-contractions have been shown to be minimal [41]. Reduction of IAP is a more logical intervention, as gravity has been shown to affect POP symptoms [42]. However, just as for hypopressive exercises, there is a strong need for more research to examine the short-term and long-term effects of inversion exercises on POP.

To date there is 1A level/recommendation for PFMT as first-line treatment for POP [1, 2]. No adverse effects have been reported from PFMT. The results from RCTs on the effect of other exercise programs besides PFMT for POP reported here are not convincing, as the studies are biased in terms of both methodological and interventional factors. McKinlay [43] referred to the seven stages of a medical intervention:

1. Promising report, clinical observation, case report or short clinical series
2. Professional and organizational adoption of the innovation
3. The public accepts the innovation—the state or thirdy party pays for it
4. The intervention becomes a standard procedure—into textbooks (still no critical evaluation)
5. RCT showing no or minimal effect
6. Professional denunciation
7. Erosion of professional support and discredit

Bø and Herbert [44] used this model to debate how new physical therapy interventions develop following the same pattern. Although physical therapy usually has no or minimal adverse effects, it is time consuming and costly for the patients, physical therapists, and society. Therefore, the authors [44] suggested a model for implementation of new interventions in physical therapy where they named the first three stages (1. Clinical observation/laboratory studies, 2. Clinical exploration, 3. Pilot studies) for the development phase. During this phase the patients must be fully informed that there is no evidence for the treatment and consent to try it out. The fourth stage is the testing phase (second phase) where the effect of the new treatment is evaluated in a high-quality RCT. If the results show meaningful clinical effect sizes, there should be a fifth stage with further refinement of the treatment with additional RCTs and studies of dose–response issues. Then, if the results are robust, with similar and additional high effect sizes, the implementation phase (third phase) with active dissemination can start; development of clinical guidelines, running of professional courses and implementation in graduate and postgraduate curricula. As PFMT is effective in the treatment of POP, we recommend this conservative approach before implementing other exercise programs. This does not mean that we discourage physical activity or other exercise programs for women with POP. On the contrary, we encourage women of all ages to continue or commence other physical activities for overall health and wellbeing. These exercise programs could be in conjunction with PFMT for POP.

The strength of the present review is the comprehensive search strategy and inclusion of RCTs only. RCT is the recommended research design for studies on cause–effect as it controls for most of the threats to internal validity; whether an effect is caused by the intervention and not external factors such as concomitant history, maturity, instrumentation, learning effect from multiple testing, selection bias, and regression towards the mean [45]. The RCT design does not control for instrumentation, experimental mortality or expectation, and these factors must be handled in addition to the randomization. All studies included in the present review except one [11] were assessor blinded and the studies control for assessor expectations. Furthermore, the trials had low drop-out and high adherence rates and used assessment methods of PFM variables and outcome measures found to be reliable and valid [46].

Limitations are the small number of studies, relatively small sample sizes and huge heterogeneity in the use of outcome measures, exercise programs and dosage of training with multiple modalities in one intervention not warranting a meta-analysis. In addition, we included RCTs published in English only. The author group of the present study included researchers representing many different countries and languages and were therefore able to search, retrieve, and read publications in other languages besides English. No full publications or abstracts written in other languages were retrieved from our searches.

## Conclusion

Based on the seven published RCTs on other exercise programs compared with PFMT or in addition to PFMT, no recommendation can be made regarding hypopressive, yoga, and inversion hip exercises for POP. PFMT remains the first-line treatment of POP. Mechanism of action for these other exercise programs should be further investigated.

## References

[1] Dumoulin C, Adewuyi T, Booth J, Abrams P, Cardozo L, Wagg A, Wein A (2017). Adult conservative management. 6th International Consultation on Incontinence. ICI-ICS.

[2] Bø K, Anglès-Acedo S, Batra A (2022). International urogynecology consultation chapter 3 committee 2; conservative treatment of patient with pelvic organ prolapse: pelvic floor muscle training. Int Urogynecol J.

[3] Bø K (2004). Pelvic floor muscle training is effective in treatment of female stress urinary incontinence, but how does it work?. Int Urogynecol J Pelvic Floor Dysfunct.

[4] Bø K (2020). Physiotherapy management of urinary incontinence in females. J Physiother.

[5] Braekken IH, Majida M, Engh ME, Bo K (2009). Test-retest reliability of pelvic floor muscle contraction measured by 4D ultrasound. Neurourol Urodyn.

[6] Peschers UM, Fanger G, Schaer GN, Vodusek DB, DeLancey JO, Schuessler B (2001). Bladder neck mobility in continent nulliparous women. BJOG.

[7] Miller JM, Ashton-Miller JA, DeLancey JO (1998). A pelvic muscle precontraction can reduce cough-related urine loss in selected women with mild SUI. J Am Geriatr Soc.

[8] Miller JM, Perucchini D, Carchidi LT, DeLancey JO, Ashton-Miller J (2001). Pelvic floor muscle contraction during a cough and decreased vesical neck mobility. Obstet Gynecol.

[9] Brækken IH, Majida M, Engh ME, Bø K (2010). Morphological changes after pelvic floor muscle training measured by 3-dimensional ultrasonography: a randomized controlled trial. Obstet Gynecol.

[10] Resende APM, Bernardes BT, Stupp L, Oliveira E, Castro RA, Girao M, Sartori MGF (2019). Pelvic floor muscle training is better than hypopressive exercises in pelvic organ prolapse treatment: an assessor-blinded randomized controlled trial. Neurourol Urodyn.

[11] Sweta K, Godbole A, Prajapati S, Awasthi HH (2021). Assessment of the effect of Mulabandha yoga therapy in healthy women, stigmatized for pelvic floor dysfunctions: a randomized controlled trial. J Ayurveda Integr Med.

[12] Pace LA, Herbert AS, Malik RD (2021). Characteristics of pelvic organ prolapse content available on social media. Neurourol Urodyn.

[13] Excellence NIfHaC. NICE guideline. Urinary incontinence and pelvic organ prolapse in women: management [NG123]. 2019. 31211537

[14] Bø K, Frawley H, Bø K, Berghmans B, Mørkved S, Van Kampen M (2015). Pelvic floor muscle training in prevention and treatment of pelvic organ prolapse. Evidence based physical therapy for the pelvic floor. Bridging science and clinical practice.

[15] Sherrington C, Herbert RD, Maher CG, Moseley AM (2000). PEDro. A database of randomized trials and systematic reviews in physiotherapy. Man Ther.

[16] Kamper SJ, Moseley AM, Herbert RD, Maher CG, Elkins MR, Sherrington C (2015). 15 years of tracking physiotherapy evidence on PEDro, where are we now?. Br J Sports Med.

[17] Cashin AG, McAuley JH (2020). Clinimetrics: Physiotherapy Evidence Database (PEDro) Scale. J Physiother.

[18] Maher CG, Sherrington C, Herbert RD, Moseley AM, Elkins M (2003). Reliability of the PEDro scale for rating quality of randomized controlled trials. Phys Ther.

[19] Macedo LG, Elkins MR, Maher CG, Moseley AM, Herbert RD, Sherrington C (2010). There was evidence of convergent and construct validity of Physiotherapy Evidence Database quality scale for physiotherapy trials. J Clin Epidemiol.

[20] Haylen BT, de Ridder D, Freeman RM (2010). An International Urogynecological Association (IUGA)/International Continence Society (ICS) joint report on the terminology for female pelvic floor dysfunction. Int Urogynecol J.

[21] Bø K, Frawley HC, Haylen BT (2017). An International Urogynecological Association (IUGA)/International Continence Society (ICS) joint report on the terminology for the conservative and nonpharmacological management of female pelvic floor dysfunction. Int Urogynecol J.

[22] Frawley H, Shelly B, Morin M (2021). An International Continence Society (ICS) report on the terminology for pelvic floor muscle assessment. Neurourol Urodyn.

[23] Hagen S, Glazener C, McClurg D (2017). Pelvic floor muscle training for secondary prevention of pelvic organ prolapse (PREVPROL): a multicentre randomised controlled trial. Lancet.

[24] Resende AP, Stupp L, Bernardes BT (2012). Can hypopressive exercises provide additional benefits to pelvic floor muscle training in women with pelvic organ prolapse?. Neurourol Urodyn.

[25] Bernardes BT, Resende AP, Stupp L (2012). Efficacy of pelvic floor muscle training and hypopressive exercises for treating pelvic organ prolapse in women: randomized controlled trial. Sao Paulo Med J.

[26] Navarro-Brazález B, Prieto-Gómez V, Prieto-Merino D, Sánchez-Sánchez B, McLean L, Torres-Lacomba M (2020). Effectiveness of hypopressive exercises in women with pelvic floor dysfunction: a randomised controlled trial. J Clin Med.

[27] Sweta KM, Godbole A, Awasthi HH, Pandey U (2018). Effect of Mula Bandha yoga in mild grade pelvic organ prolapse: a randomized controlled trial. Int J Yoga.

[28] Gorji Z, Pourmomeny AA, Hajhashemy M (2020). Evaluation of the effect of a new method on the pelvic organ prolapse symptoms. Low Urin Tract Symptoms.

[29] Caufriez M (1997). Gymnastique abdominale hypopressive.

[30] Vicente-Campos D, Sanchez-Jorge S, Terron-Manrique P (2021). The main role of diaphragm muscle as a mechanism of hypopressive abdominal gymnastics to improve non-specific chronic low back pain: a randomized controlled trial. J Clin Med.

[31] Martín-Rodríguez S, Bo K (2019). Is abdominal hypopressive technique effective in the prevention and treatment of pelvic floor dysfunction? Marketing or evidence from high-quality clinical trials?. Br J Sports Med.

[32] Hernández RRV (2018). Efficacy of hypopressive abdominal gymnastics in rehabilitating the pelvic floor of women: a systematic review. Actas Urol Esp (Engl Ed).

[33] Moreno-Muñoz MDM, Hita-Contreras F, Estudillo-Martinez MD (2021). The effects of abdominal hypopressive training on postural control and deep trunk muscle activation: a randomized controlled trial. Int J Environ Res Public Health.

[34] Resende AP, Torelli L, Zanetti MR (2016). Can abdominal hypopressive technique change levator hiatus area?: a 3-dimensional ultrasound study. Ultrasound Q.

[35] Navarro Brazález B, Sánchez Sánchez B, Prieto Gómez V, De La Villa PP, McLean L, Torres Lacomba M (2020). Pelvic floor and abdominal muscle responses during hypopressive exercises in women with pelvic floor dysfunction. Neurourol Urodyn.

[36] Reman T, Cacciari L, Voelkl JG, Malcolm D, Budgett D, Kruger J, Dumoulin C. Intravaginal pressure profile during two diaphragmatic aspiration tasks in women with stress urinary incontinence: a cross sectional study. Neurourol Urodyn. 2020;39(S2).

[37] Bø K, Hagen RH, Kvarstein B, Jørgensen J, Larsen S, Burgio KL (1990). Pelvic floor muscle exercise for the treatment of female stress urinary incontinence. III. Effects of two different degrees of pelvic floor muscle exercises. Neurourol Urodyn.

[38] Dumoulin C, Morin M, Danieli C (2020). Group-based vs individual pelvic floor muscle training to treat urinary incontinence in older women: a randomized clinical trial. JAMA Intern Med.

[39] Bø K, Talseth T, Holme I (1999). Single blind, randomised controlled trial of pelvic floor exercises, electrical stimulation, vaginal cones, and no treatment in management of genuine stress incontinence in women. BMJ.

[40] Due U, Brostrom S, Lose G (2016). Lifestyle advice with or without pelvic floor muscle training for pelvic organ prolapse: a randomized controlled trial. Int Urogynecol J.

[41] Kruger J, Budgett D, Goodman J, Bo K (2019). Can you train the pelvic floor muscles by contracting other related muscles?. Neurourol Urodyn.

[42] Haylen BT, Maher CF, Barber MD (2016). An International Urogynecological Association (IUGA)/International Continence Society (ICS) joint report on the terminology for female pelvic organ prolapse (POP). Int Urogynecol J.

[43] McKinlay JB (1981). From "promising report" to "standard procedure": seven stages in the career of a medical innovation. Milbank Mem Fund Q Health Soc.

[44] Bø K, Herbert RD (2009). When and how should new therapies become routine clinical practice?. Physiotherapy.

[45] Thomas JR, Silverman S, Nelson J (2015). Experimental research. Research methods in physical activity.

[46] Bø K, Sherburn M (2005). Evaluation of female pelvic-floor muscle function and strength. Phys Ther.

